# Mesenchymal Stem Cell-Derived Extracellular Vesicles in Tendon and Ligament Repair—A Systematic Review of In Vivo Studies

**DOI:** 10.3390/cells10102553

**Published:** 2021-09-27

**Authors:** Victor Lu, Maria Tennyson, James Zhang, Wasim Khan

**Affiliations:** 1 School of Clinical Medicine, University of Cambridge, Cambridge CB2 0SP, UK; victorluwawa@yahoo.com.hk (V.L.); zz357@cam.ac.uk (J.Z.); 2 Department of Trauma and Orthopaedic Surgery, Addenbrooke’s Hospital, University of Cambridge, Cambridge CB2 0QQ, UK; maria.tennyson@addenbrookes.nhs.uk

**Keywords:** mesenchymal stem cells, extracellular vesicles, tendon, ligament, collagen, biomechanics, macrophage

## Abstract

Tendon and ligament injury poses an increasingly large burden to society. This systematic review explores whether mesenchymal stem cell-derived extracellular vesicles (MSC-EVs) can facilitate tendon/ligament repair in vivo. On 26 May 2021, a systematic search was performed on PubMed, Web of Science, Cochrane Library, Embase, to identify all studies that utilised MSC-EVs for tendon/ligament healing. Studies administering EVs isolated from human or animal-derived MSCs into in vivo models of tendon/ligament injury were included. In vitro, ex vivo, and in silico studies were excluded, and studies without a control group were excluded. Out of 383 studies identified, 11 met the inclusion criteria. Data on isolation, the characterisation of MSCs and EVs, and the in vivo findings in in vivo models were extracted. All included studies reported better tendon/ligament repair following MSC-EV treatment, but not all found improvements in every parameter measured. Biomechanics, an important index for tendon/ligament repair, was reported by only eight studies, from which evidence linking biomechanical alterations to functional improvement was weak. Nevertheless, the studies in this review showcased the safety and efficacy of MSC-EV therapy for tendon/ligament healing, by attenuating the initial inflammatory response and accelerating tendon matrix regeneration, providing a basis for potential clinical use in tendon/ligament repair.

## 1. Introduction

Tendon and ligament injuries make up about 50% of all musculoskeletal injuries and cost $30 billion a year to manage [1]. These can be due to underlying tendon diseases, such as inflammatory or degenerative changes seen in tendinopathies, or due to acute traumatic injury. Tendons generally have limited vascularisation when compared to muscle, especially at the tendon–bone interface. There is reduced angiogenesis at these sites due to inhibitory factors such as endostatin, secreted from neighbouring cells [2]. The blood supply to tendons generally comes from three sources: the myotendinous junction, the osteotendinous junction, and the tendon sheath. Ligaments, on the other hand, have blood supply from the synovium as well as from the surrounding soft tissue. The healing response in ligaments can be divided into the hemorrhagic, inflammatory, reparative, and remodelling phases. The final phase can take up to months or years to complete, and is subject to a number of external factors, with minimal exercise, smoking, and high cholesterol associated with poorer outcomes [3]. These injuries can be severely debilitating, whether in athletes trying to return to their high level of sporting ability, or patients returning to independent living. Furthermore, a suboptimal healing process can cause scar tissue formation, increasing the chance of reinjury [4].

Traditional methods of tendon and ligament repair include suture anchors [5,6] and autogenous tendon grafts [7]. When tendons are divided, the ends can be approximated and sutured to promote healing. Complications such as adhesions [8], repair site gap formation [9], or chondrolysis [10] may occur. Tissue grafting is another well-established method of tendon repair. Autograft involves harvesting tissue from the patient’s own body to replace the damaged tissue, and despite the success rate, complications such as donor site morbidity issues or lack of adequate tissue may arise. Allograft and xenograft involve tissue transplantation from a human or animal, respectively. These have greater availability and flexibility but risk rejection, disease transmission, and zoonotic transmission [11].

In recent years, developments of stem cell therapies have created new avenues for treatment [12]. Induced pluripotent (iPSC) and embryonic stem cells (ESCs) have the highest differentiation capacity and flexibility; however the unlimited regenerative nature of these cells can potentially increase the risk of teratoma development [13] or ectopic bone formation [14]. Mesenchymal stem cells (MSCs) do not have the ethical concerns that surround the use of ESCs but can potentially influence tumorigenesis and lead to cancer treatment resistance [11]. These cells can be used in isolation or with appropriate biomaterial using a suitable scaffolding technique to create the most appropriate environment for tissue regeneration. The purpose of an ideal scaffold should be to seamlessly transition from the artificial alignment of the damaged tissue to the natural regeneration of native tissue using the body’s inherent processes [15].

Recently, there has been a shift in the approach to cell-free therapy, with extracellular components shown to have similar regenerative properties without the potentially harmful effects of entire cell transplantations [16]. Extracellular vesicles (EVs) are extracellular lipid membrane–bound particles that contain host cell-derived protein or nucleic acid messengers, and have an effect on target cells via paracrine or autocrine regulatory functions [17]. This new type of therapy has the potential to change the microenvironment of healing tissue, reducing the inflammatory process and promoting regeneration, as shown in various studies across different organ systems, such as neural [18], musculoskeletal [19], and cardiac [20] tissues. This systematic review explores whether mesenchymal stem cell-derived extracellular vesicles (MSC-EVs) can facilitate tendon/ligament repair in vivo.

## 2. Methodology

This review was carried out according to the 2020 Preferred Reporting Items for Systematic Reviews and Meta Analyses (PRISMA) statement protocol [21]. An initial review of the literature was performed to gauge the heterogeneity of the literature, after which our search criteria were formulated. This lowered the chance that important studies would be missed.

On 26 May 2021, a systematic search was performed on Embase, Medline, Web of Science, and Cochrane Library, which were considered comprehensive. No filters of any sort were used, and databases were searched from conception. The search strategy is shown in Supplementary Table S1. All studies found by our search were imported into Mendeley and deduplicated. VL and MT independently completed title and abstract screening and agreement between authors was assessed and generated 93% agreement. A third reviewer (WK) was contacted for unresolvable disagreements. Next, full-text screening was performed by VL and MT, based on the inclusion and exclusion criteria shown in Supplementary Table S2. Again, a third reviewer (WK) was consulted for any disagreements. A ‘snowball’ search was then performed on 2 June 2021, whereby references of the included studies as well as studies that cited any of the included studies were independently searched by VL and MT, using Google Scholar to identify and screen studies. Studies that performed in vivo experiments, using EVs isolated from human– or animal–derived MSCs, were included. Studies that characterised their MSC population using guidelines from the International Society for Cellular Therapy [22] and characterised their EV population using International Society for Extracellular Vesicles (ISEV) standards were included [23]. In vitro, ex vivo, and in silico studies were excluded, and studies without a control arm were excluded.

Data extraction was independently performed by VL and MT, with a third reviewer (WK) to resolve disagreements. Data were extracted into data tables created in a standardised excel spreadsheet for assessment of study quality and evidence synthesis. Data from each study were split into 4 categories:

1. Isolation and characterisation of MSCs, including source of MSCs, cellular origin, cell treatment to extract MSCs, and procedures to verify MSCs (e.g., flow cytometry, western blotting).
2. Characterisation and purification of EVs, including MSC purification to extract EVs, EV dimensions, EV biomarkers, imaging used to visualise EVs, and EV active component.
3. In vivo model, including method of EV delivery, type of in vivo model, how tendon/ligament injury was induced, animal age, animal weight, animal gender, total number of animals used per experimental group, and follow-up time.
4. In vivo findings, including macroscopic appearance, imaging results, histopathological results, biochemical findings, and biomechanical findings.

Quality assessment was carried out independently by MT and VL using the SYstematic Review Center for Laboratory animal Experimentation (SYRCLE) tool [24]. The main categories assessed were selection bias, performance bias, blinding bias, attrition bias, and reporting bias. Discrepancies were consulted with WK.

This review was prospectively registered in the International Prospec-tive Register of Systematic Reviews PROSPERO (https://www.crd.york.ac.uk/PROSPERO/display_record.php?RecordID=257333, accessed on 25 September 2021).

## 3. Results

**Figure 1:**
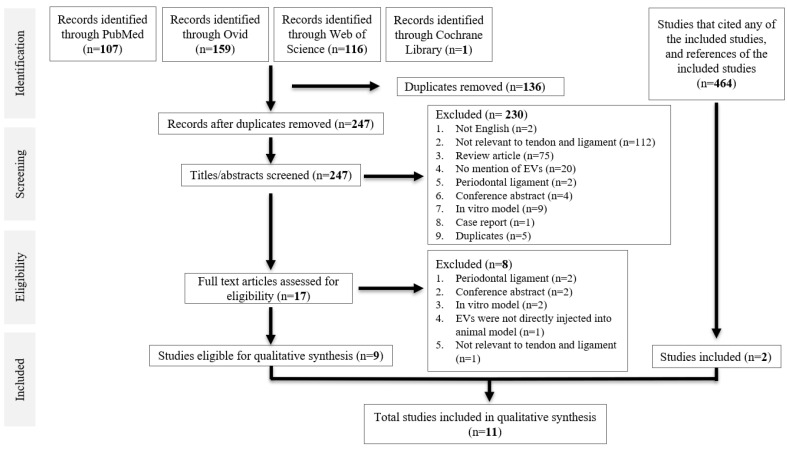
PRISMA diagram.

A total of 383 studies were identified from database searching. After de-duplication, 247 studies were identified for title and abstract screening, of which 17 full-text studies were reviewed. Nine studies were eligible for data synthesis [25,26,27,28,29,30,31,32,33]. Searching references of the included studies, as well as studies that cited any of the included studies, yielded two more studies [34,35], giving a total of 11 studies for qualitative synthesis. All were case–control studies. A PRISMA diagram is shown in Figure 1.

### 3.1. Characterisation of MSCs

The majority of studies used animal-derived MSCs rather than human-derived MSCs (Table 1). Of the seven studies that used animal-derived MSCs (involving 310 subjects), the most common MSC donor was Sprague–Dawley rats, with four studies involving 172 subjects utilising them [25,27,32,34]. One study involving 16 subjects used the Lewis rat [29], and one study involving 32 subjects used NF-κB–luciferase reporter mice [26]. This was done to investigate how MSC-EVs could alter macrophage NF-κB inflammatory signalling [26]. Of the four studies that used human-derived MSCs, involving 138 subjects [30,31,33,35], two studies involving 93 subjects obtained MSCs from the umbilical cord [30,33], one study involving 35 subjects from adipose tissue [35], and one study involving 10 subjects from bone marrow cells [31].

Regarding the culture medium, alpha-modified minimum essential medium (α-MEM) was most commonly employed, used in seven studies involving 325 subjects. Other culture methods include Dulbecco’s Modified Eagle’s Medium (DMEM) utilised in two studies involving 72 subjects [32,34], MesenCult™ Basal Medium utilised in one study involving 16 subjects [29], and serum-free medium (OriCell) utilised in one study involving 35 subjects [35].

The two most common methods for characterising MSCs were surface-maker expression using flow cytometry (used in seven studies with 310 subjects [25,26,27,28,29,32,34]), and testing for the absence of haematopoietic surface markers CD34 and CD45 (used in five studies involving 226 subjects [25,28,29,32,34]). Another, less common method was trilineage differentiation into adipocytes, osteoblasts, and chondrocytes, seen in four studies involving 140 subjects [27,29,32,34].

### 3.2. Characterisation of EVs

All studies isolated their EVs from MSCs using cell-culture media by differential centrifugation and ultracentrifugation (Table 2). Shen et al. created two EV groups, one isolated from MSCs that was primed with 100 ng/mL IFNγ at passages two to four (labelled ‘iEV’), and one isolated from MSCs without IFNγ pre-treatment [26]. This was done based on previous literature suggesting that MSC-EVs primed by inflammatory mediators could enhance their immunosuppressive functions [36]. Li et al. also created two EV groups, one derived from hydroxycamptothecin-primed human umbilical cord MSCs, and one from unprimed MSCs. The rationale was that hydroxycamptothecin elicits endoplasmic reticulum (ER) stress in MSCs, leading to increased ER stress effector proteins in secreted EVs, which increases the ability for EVs to prevent myofibroblast transformation and hence tendon adhesion [33].

The most widespread method of visualising EVs was using transmission electron microscopy (TEM) (used in nine studies involving 372 subjects [25,26,27,28,31,32,33,34,35]), and of those, three studies involving 105 subjects used the method to also measure EV dimensions [32,33,34]. Other methods of visualising EVs included atomic force microscopy (ATM), used in one study involving 16 subjects [29], and an 80kV electron microscope [30]. Additional methods for visualising EVs include tunable resistive pulse sensing (TRPS) using Izon’s qNano Gold in four studies involving 125 subjects [25,26,31,35], nanoparticle tracking analysis (NTA) with ZetaView in three studies involving 202 studies [27,28,30], and AFM in one study [29]. EVs were characterised by flow cytometry and western blotting, with CD9, CD63, TSG-101 being the most common EV markers identified. Gissi et al. attributed the increased extracellular matrix–tendon remodelling to MMP14 and pro-collagen1A2, which were identified in EVs by dot blot [29]. Yao et al. concluded that human umbilical cord-derived MSCs release low levels of miR-21a-3p, which manipulates p65 activity to inhibit tendon adhesion [30].

### 3.3. Animal Models

Rats were the most common animal model used, with ten studies involving 430 subjects using them as recipients of EVs (Table 3). Amongst rat species, Sprague–Dawley rats were the most frequently used [25,27,30,32,33,34]. The remaining study involving 18 subjects used rabbits [34]. Across all eleven studies, 448 animal subjects (range: 10–90) were used for in vivo analysis. Animal age and weight were reported in all but one study [25]. The most common site used to induce tendon/ligament damage was the Achilles tendon, whereby surgical removal was performed in six studies involving 251 subjects. Two studies involving 100 subjects surgically removed part of the patella tendon [25,27], one study involving 10 subjects transected the medial collateral ligament (MCL) at its midpoint [31], and two studies involving 89 subjects created a rotator cuff tear model, by detaching the supraspinatus at its insertion at the humerus [32,35]. Only one study involving 18 subjects used a non-surgical technique, utilising type I collagenase solution to cause tendon damage [34]. All studies injected EVs at the injury site, apart from Huang et al., who injected exosomes into the tail vein of a rotator cuff tear murine model [32]. Follow-up time ranged from 7 days to 18 weeks, with most studies sacrificing animals at different stages depending on the outcome measure to be investigated. For example, Wang et al. randomly sacrificed 7 rabbits at 6 weeks for fatty infiltration assay, but sacrificed the remaining 21 rabbits at 18 weeks for histological and biomechanical evaluation [35].

All studies were designed in a case-control format. Three studies involving 116 subjects divided their experimental subjects into two groups, a control and MSC-EV group [27,31,32]. The rest included multiple experimental groups, with one study involving 35 subjects including a sham surgery group, whereby the tendon would be exposed but not surgically manipulated [35]. One study investigating a dose-dependent relationship between EV concentration and tendon repair further separated their EVs into high (8.4 × 10^12^ EVs) and low concentrations (2.8 × 10^12^ EVs) [29]. One study utilised hydrogel to promote long-term exosome retention and encourage sustained exosome release, and hence created a separate group that only received hydrogel [28]. Shen et al. compared the efficacy of IFNγ-primed MSC-EVs versus naïve MSC-EVs and hence had three experimental groups in total [26].

### 3.4. In Vivo Findings

Six studies involving 280 subjects performed macroscopic analysis [27,28,30,31,33,35], but one only used it to look for fatty infiltration, confirming the establishment of a rotator cuff tear model (Table 4) [35]. Yu et al. showed that the appearance of the injured tendon better approximated normal tendon after exosome treatment [27]. Two studies involving 100 subjects observed reduced scar formation [28,31] and two studies involving 93 subjects reported reduced tendon adhesion to peri-tendinous tissue [30,33]. Histological analysis was performed by all studies. Five studies utilised scoring systems; Shi et al. utilised a fibre alignment score as a proxy for tendon healing [25]. The other four studies involving 161 subjects used histological scores, which includes sub-scores such as fibre structure, cellularity, vascularity, degree of adhesion [27,29,30,33]. Collagen deposition and alignment were assessed by eight studies involving 345 subjects [25,26,27,28,30,31,34,35], all of which reported more compact and regularly aligned collagen fibres in EV-treated tendons. One study utilised angiography to show that exosomes promoted angiogenesis around the injury site [32].

All studies performed biochemical analysis. Four studies involving 227 subjects explicitly mentioned that EVs reduced the expression of pro-inflammatory cytokines such as IL-1β and IL-6, and increased expression of anti-inflammatory cytokines such as IL-10 and TGF-β1 [25,28,32,35]. Shen et al. demonstrated decreased gene expression and protein expression in the tendon not performed. Three studies involving 154 subjects directly tested the impact of EVs on macrophage polarisation [28,31,32]. Huang et al. showed that exosomes decreased CD86, an M1 macrophage surface marker [32], whilst Shi et al. demonstrated that exosomes decreased iNOS+ M1 macrophages and increased Arg1+ M2 macrophages [28], despite the former being done in vitro. However, Chamberlain et al. reported that exosome treatment had no significant effect on M1 or M2 macrophage number, whilst EV-educated macrophages (made by exposing CD14^+^ macrophages to MSC-EVs) decreased endogenous M1/M2 macrophage ratio [31]. Virtually all studies reported increased expression of genes related to collagen and tendon matrix formation, such as COL1a1, COL2a1, COL3a1, SCX, Sox9. Gissi et al. also reported a more favourable collagen ratio after EV treatment, i.e., increased collagen type I and decreased collagen type III expression [29].

Eight studies performed biomechanical analysis. In a bilateral rotator cuff tear model, Wang et al. found that the mean ultimate load to failure in the MSC-EV treated group was significantly greater (132.7 N versus 96 N) than in the control group [35]. In a murine patellar tendon injury model, Yu et al. noticed that stress at the failure of the regenerated tendons and Young’s modulus were 1.84-fold and 1.86-fold higher in the MSC-EV treated group than controls [27]. Most other studies reported that EV treatment increased the maximum stiffness, breaking load, and tensile strength of regenerated tendons; however, three studies reported no significant difference in biomechanical properties between EV-treated and control groups [30,31,33].

### 3.5. Risk of Bias

**Figure 2:**
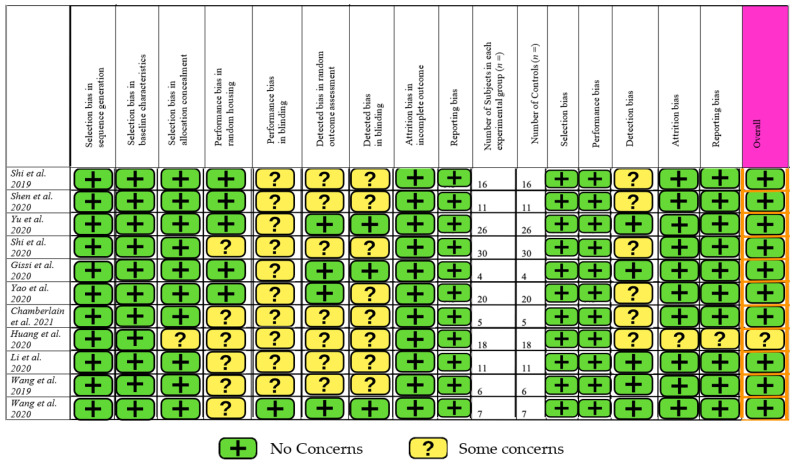
Risk of bias assessment.

The SYRCLE risk of bias tool for animal studies was used, containing 15 different parameters [24]. A summary of this is provided in Figure 2. Ten studies had a low level of concern overall, but one study had some concern about the risk of bias [32]. The main contributors to bias were blinding and detection bias. There was little selection and reporting bias. Overall, the studies included in this review are of high quality with a low risk of bias.

## 4. Discussion

Over the past 30 years, MSCs have become a cornerstone of tissue engineering and biotechnology research, having been used in a variety of scenarios, such as evaluating the optimum cell dose for treatment of non-union bone fractures [37] and evaluating the capacity of MSCs for managing osteochondral defects [38]. There has recently been increasing interest in the use of MSCs and their derived EVs for tendon and ligament repair, presenting the need for a review of the current literature in this field.

All studies in this review reported better tendon/ligament repair following treatment with MSC-EVs. A variety of outcome measures were employed in each study to examine the impact of EVs on tendon/ligament repair, but not all studies found improvements in every parameter measured. All 448 subjects were treated with MSC-EVs without immunogenic or significant complications.

### 4.1. MSC Isolation, Differentiation, and Culture Media

Current protocols for MSC isolation from bone marrow rely on density gradient centrifugation (DGC) or size-exclusion chromatography (SEC) [39,40,41]. Studies have demonstrated that EV isolated from stem cell culture by ultrafiltration followed by SEC results in a higher yield while preserving EV biophysical and functional properties [42]. All the included studies in this review only used several ultracentrifugation steps without SEC. Obtaining high yields of MSCs is crucial for their clinical application. Improvements on the current isolation protocol, e.g., the isolation of MSCs using a Good Manufacturing Practice (GMP)–compatible fabric filter system device resulted in a higher yield of colony–forming units fibroblasts (CFU-F), producing substantially more MSCs with a similar subpopulation composition and functional characteristics as MSCs isolated by DGC [43].

Culturing autologous MSCs in different media and conditions has been shown to affect growth characteristics, surface marker distribution and chondrogenic differentiation [44]. The media used was DMEM or αMEM with varied concentrations of foetal calf serum +/− additives, e.g., PDGF, FGF-2, and ascorbic acid. This study found that chondrogenic differentiation was superior in a medium composed of DMEM with low glucose, 10% foetal calf serum, and 1% penicillin/streptomycin (referred to as medium A) [44]. Sotiropoulou et al. showed that expansion media, growth factors, plating density and flask manufacturer have an impact on MSC characteristics [45]. Some studies have demonstrated inferior chondrogenic differentiation results of MSCs expanded in a control medium when compared to embryonic stem cell medium [44,46]. In the absence of a standardised culture protocol designed to maximise the chondrogenic differentiation of MSCs, there will be continued variation between studies. Included studies utilised α-MEM, DMEM, serum-free OriCell, and MesenCult^TM^; the type chosen did not preclude MSC’s ability to undergo trilineage differentiation and express important cell surface markers such as CD44^+^ and CD90^+^. Nevertheless, only MSCs that were cultured in DMEM were CD3^−^ [34], and MSCs cultured in MesenCult^TM^ Basal Medium were CD29^+^ [31]. α-MEM is one of the most commonly used media, which contains higher concentration of essential nutrients than its predecessor, basal medium eagle (BME). Sotiropoulou et al. reported that culture media based on α-MEM are more suitable for the expansion and proliferation of multipotent MSCs [45]. Only Chamberlain et al. added l-glutamine to the media [31] to encourage MSC growth. Nevertheless, Sotiropolou et al. suggested that Glutamax better supports MSC growth. Reasons were two-fold: (1) the presence of dipeptide l-alanyl-l-glutamine in Glutamax; (2) metabolism of glutamine leads to ammonia, which restricts MSC growth [47].

A number of studies compared the differentiation ability between MSCs derived from different species and different sources. Scuteri et al. found that rat MSCs had a greater chondrogenic and osteogenic differentiation potential than human MSCs, whereas human MSCs had a greater adipogenic differential potential [48]. Scuteri et al. also noted variation in differentiation time among human MSCs [48]. Martínez-Lorenzo et al. showed that rabbit and sheep MSCs were able to differentiate into chondrocytic lineages more easily than human MSCs [49]. Human MSCs from different sources display certain differences despite meeting the minimal criteria of MSC characterization [50]. All seven of the included studies that utilised animal-derived MSCs were able to undergo trilineage differentiation into adipocytes, osteoblasts, and chondrocytes. However, the four studies that used human MSCs did not report results of MSC differentiation, perhaps hinting reporting bias if their MSCs performed poorly regarding differentiation [30,31,33,35].

Studies also attempted to identify markers associated with better MSC properties. Nagano et al. demonstrated that human umbilical cord MSCs with a high ALDH activity proliferated more than those with low ALDH activity [51]. Coipeau et al. suggested that age and sex of the donors influence the differentiation process and found that trabecular bone MSCs from elderly patients are not a good starting material for cell therapy usage for bone repair and regeneration, unless cultured in the presence of FGF-2 [52]. This suggests a possible role for allogenic MSC-EVs. Seven included studies added penicillin and streptomycin to their culture medium [26,27,28,29,32,33,34] to avoid bacterial contamination. However, in vitro studies have shown that antibiotics can modify gene expression via drug-dependent gene regulatory elements [53]. Furthermore, Ryu et al. directly identified a group of differentially expressed genes in HepG2 cells, that had significantly enhanced expression in response to penicillin/streptomycin [54]. This suggests that data from studies that utilise antibiotics in cell culture should be examined with caution.

All included studies supplemented their culture with foetal bovine serum (FBS), apart from Wang et al., who cultured MSCs in a serum-free medium (OriCell) [35]. FBS contains growth factors and macromolecular components essential for MSC growth. Nevertheless, evidence suggests that it may be time to step away from FBS, not only due to issues such as xenoimmunisation and contamination from viral or prion particles [55], but also because of proven benefits from other serum supplements. Bovine serum albumin with insulin-transferrin-sodium selenite better augmented protein expression levels in bovine embryos than FBS [56]. GMP-compliant serum-free or xeno-free media are potential alternatives. Adipose stem cells (ASCs) expanded in xeno-free media with significant higher doubling rates than in FBS (*p* < 0.001) [57]. No included studies used human serum, which could create an environment more akin to that of humans. Shahdadfar et al. showed that autologous human serum provides ASCs with a more rapid expansion and better genomic stability than FBS [58].

### 4.2. EV Isolation and Administration

The choice of EV isolation procedure significantly impacts the EV yield from serum. Brennan et al. highlighted challenges and limitations in isolating EVs from small volumes of bio-banked serum [59]. The abundance of the EV marker CD63, detected by western blot, correlated positively with the amount of protein in the sample and not the nanoparticle tracking analysis (NTA) particle counts. Flow cytometry detection of CD63 is an alternative approach to confirm EVs. It is likely that NTA overestimates the amount of EVs due to the presence of protein aggregates and lipoproteins, which are often present in EV isolate due to shared physical properties [60]. This highlights a limitation in using particles/ug to determine EVs/ug when dealing with human serum. Resolution and accuracy are low for particles with diameters <250 nm as multi-Gaussian fittings are often not obvious, meaning that the size range of NTA is limited [61]. Eight of the included studies used CD63 as an EV marker, with only Yu et al., Shi et al., and Gissi et al. opting not to use this [27,28,29]. Three studies in this review [27,28,30] used NTA; hence, the EV yield could be lower than other included studies. Strategies are being developed to obtain highly pure EVs from plasma and serum without lipoprotein or soluble protein contamination e.g., using a combination of differential ultracentrifugation and size exclusion chromatographyin tandem [62].

Different lipoproteins co-purified with EVs might promote pro or anti-inflammatory responses. OA chondrocytes express receptors for oxidised low-density lipoprotein, which promotes pro-inflammatory macrophage differentiation, and is implicated in OA disease progression [63]. Conversely, high density lipoproteins can bind pro-inflammatory miRNAs [64]. Although there have not been studies demonstrating the implications of co-isolated lipoproteins on tendon and ligament healing, further work must be done to characterise any associated effects on ligament healing. This is particularly relevant, as many of the included studies did not characterise the active EV component. Only two studies identified their active components as Pro-collagen1A2 and MMP-14 [29] and MicroRNA-21-3p [30], respectively.

The preferred application of exosomes was an intra-articular injection in aqueous solution i.e., PBS or saline [29,30,32,35] (please refer to Table 3, method of delivery). Two studies used fibrin gel [25,27], one an alternative hydrogel [28] and another a collagen gel as their delivery vehicle [26]. Fibrin gel is a biologically derived and FDA-approved hydrogel, favourable for cell adhesion and infiltration [65]. Yu et al. utilized fibrin gel as the carrier of bone marrow-derived stem cell exosomes (BMSCs-exos), which was well retained in the defect area, released into tissue, and internalized by local CD146^+^ TSPCs [27]. They used lower doses of exosomes than previous studies based on a rat model [66] and attributed their positive results to the fibrin gel’s controlled-release of EVs to the target site, so that the concentration of exosomes in the injury area was likely to be higher than those applied systematically.

Whilst all studies directly injected EVs intra-articularly, the dose delivered varied and is not always reported. Chamberlain et al.’s Achilles’ tendon injury model used 1 × 10^6^ EVs in their study, which is much lower than that used by Gissi et al. [29,31]. Gissi et al.’s rat Achilles tendon injury model study data suggest that EV treatment accelerates the progression of healing in the remodelling stage of tendon repair in a dose-dependent manner [29]. Higher concentrations of MSC-EVs (8.4 × 10^12^ EVs in 50 μL PBS) performed better than lower concentrations (2.8 × 10^12^ EVs in 50 μL PBS) and MSCs alone. Shi et al. did not provide a concentration but did provide a weight of 25 µg BMSC-EVs [25], which could contain variable amounts of lipoproteins contributing to the mass. Determining the optimum dose of EVs is difficult since different animal models and different injury sites were used; this should be a topic for further study.

### 4.3. Modifying EVs to Enhance their Biological Function

There are many strategies that can modify bioactive molecules to enhance their treatment effectiveness, such as the active loading of EVs with short interfering RNAs (siRNAs) via electroporation [67] and the genetic modification of human adipose-derived stem cells (hASCs) to load miR-375 into hASC-derived exosomes to improve osteogenic differentiation [68]. Huang et al. pre-treated MSCs with atorvastatin to increase their cardioprotective function. This resulted in lower cardiomyocyte apoptosis and greater angiogenesis in rat models of acute myocardial infarction [32]. Wang et al. performed cyclic stretch on human periodontal ligament cells resulting in the secretion of exosomes that were better at inhibiting proinflammatory IL-1β secretion from macrophages [34]. Li et al. pre-treated human umbilical MSCs with hydroxycamptothecin, resulting in a greater suppression on fibroblast proliferation and a better extrinsic fibrotic tendon repair, paving the way for improved natural intrinsic tenocyte regeneration [33]. Studies suggest that pro-inflammatory stimuli increase the immunosuppressive functions of MSC-EVs [69,70]. Shen et al. found that IFNγ-primed MSCs produced EVs that can better reduce NF-κB activity and the subsequent expression of pro-inflammatory mediators in injured tenocytes, and promote macrophage differentiation into the anti-inflammatory M2 phenotype [26]. A similar effect has also been reported in in vivo models of cartilage injury, with IFNγ-stimulated MSCs enhancing chondrogenesis [71].

#### Role of MMP-14 and miR-21 in Tendon/Ligament Repair

The tendon regeneration capabilities of MSC-EVs have been ascribed to various active components that they secrete. Gissi et al. partly attributed the increased tendon healing in a rat Achilles tendon injury model to pro-collagen1A2 and MMP-14 expression in EVs derived from rat bone marrow MSCs (rBMSCs-EVs) [29]. Gulotta et al. suggested that MMP-14, which plays a role in tendon-bone insertion site formation during embryogenesis, is responsible for increased biomechanical strength and fibrocartilage presence at tendon–bone insertion sites [72]. In a complete flexor tendon laceration rat model, Oshiro et al. noted that MMP-14 levels steadily increased in the intermediate to later stages of tendon healing [73]. This suggests that in addition to its key role during embryogenesis [74], MMP-14 is necessary for the remodelling phase of tendon healing. The exact mechanism is unknown; however, some studies suggest that MMP-14 is a key player in the cell surface activation of MMP-2 [75], MMP-9, and MMP-13 [73], resulting in tissue remodelling. Mechanistically, tendon repair via MSC-derived MMP-14 could happen by increased degradation of weaker fibrotic tissue, as suggested by increased cardiac fibroblast degradation after MSC treatment in a rat model of post-ischaemic heart failure [76]. Furthermore, studies have shown that MMP-14 is a collagenolytic enzyme that plays a crucial role in collagen homeostasis, whose inhibition in a mice MMP-14 knockout model led to a fibrosis-like phenotype [77]. MMP-14 could also trigger COX-2 expression, as shown in a study using U87 glioma cells [78]. COX-2 inhibition has been shown to be detrimental to healing at the tendon-to-bone interface in a study where the tendon healing failure rate was significantly higher in rats treated by NSAIDs than in the control group [79]. Thus, it is possible that MMP-14 re-establishes a niche that is similar to native tissue, and conducive to tendon healing.

The beneficial effects of MSC-EVs have also been ascribed to the decreased secretion of mediators. miR-21 is a known regulator of tissue fibrosis. A recent study used high-throughput miRNA sequencing to show that miR-21a-5p was highly enriched in macrophage exosomes and promoted tendon adhesion via Smad7 expression [80]. With this theoretical basis, Yao et al. sequenced human umbilical MSCs (HUMSCs) and their derived exosomes and found that miR-21a-3p was among the most highly expressed [30]. In vivo studies showed that HUMSCs-derived exosomes had a lower expression of miR-21a-3p than HUMSCs, leading to reduced TGF-β1-induced fibroblast proliferation. Excessive extracellular matrix deposition, as indicated by increased collagen III and α-SMA expression, are pathognomonic for fibrotic disease. The lower expression of miR-21a-3p in HUMSCs-derived exosomes resulted in significantly lower collagen III and α-SMA expression, consistent with the conclusion that a low abundance of miR-21 activity leads to decreased fibrosis and tendon adhesion [30].

### 4.4. EV Educated Macrophages

There is evidence that a main path by which MSC-EVs contribute to tendon and ligament repair is through effects on other cells, with macrophages being important targets. These macrophages are known as EV-educated macrophages (EEVs). It is well known that macrophages play a key role in all stages of tissue repair. Regarding tendon healing, biomechanical testing demonstrated that macrophage metalloelastase-deficient mice had a lower ultimate force and stiffness and a decreased level of type I pro-collagen mRNA compared to wild-type mice [81]. In vivo macrophage depletion using clodronate liposomes during the early healing process (day 5) limited granulation tissue formation and comprised final ligament strength, even though macrophage levels returned to normal after day five [82]. de la Durantaye et al. injected mice four hours prior to tenotomy with clodronate liposomes, with daily injections until four days post-surgery, and found a decreased extracellular matrix formation and cell proliferation compared to PBS-treated mice [83]. However, it was reported that clodronate-treated mice had greater Young’s modulus and maximal stress than PBS-treated mice. The superior ultimate tensile stress in clodronate-treated mice appears to contradict the findings of Schlundt et al. [84]. Yet, this could be explained by the fact that clodronate treatment was limited to four days post-surgery, thus mainly affecting M1 macrophage levels, which is the first subset to invade tissues. After an injury, an acute inflammatory reaction is usually accompanied by extensive M1 macrophage infiltration, which secretes pro-inflammatory mediators (TNF-α, IL-6, IL-1β) to increase vessel wall permeability, recruit leukocytes and fibroblasts [85]. M1 macrophages have also been reported to inhibit chondrogenesis, exacerbate experimental osteoarthritis [86] and inhibit the tenogenic differentiation of tendon-derived stem cells [87]. Afterwards, macrophages are polarised into the M2 phenotype which secrete anti-inflammatory cytokines (TGF-β, IL-10) for inflammation resolution and repair. However, excessive M1 macrophage activity encourages excessive fibroblast activity, leading to scar tissue formation. This hampers tissue remodelling at tendon–bone interfaces and fibrocartilage repair, whilst increasing fibrosis and tendon adhesion [88]. This suggests that excessive M1 macrophage activity is likely responsible for the inferior mechanical properties of Achilles tendons in the study by de la Durantaye et al. [83].

Shi et al. used an Achilles tendon murine model and showed that seven days after tenotomy, bone marrow MSC-derived exosomes (BMSC-exos) increased Arg1+ M2 macrophages and decreased iNOS+ M1 macrophages [28]. In an in vitro study, Chamberlain et al. generated EEVs, which were M2-like macrophages, by exposing CD14^+^ macrophages to EVs [31]. The functional benefits conferred by an increased M2:M1 macrophage ratio was shown macroscopically by reduced scar hyperplasia in the BMSC-exos treated group and histologically by an increased Safranin O-positive area, suggesting increased regeneration at the tendon–bone interface. Finally, the BMSC-exos treated group enjoyed increased maximum force and Young’s modulus compared to controls, with no significant difference with the normal group [28]. Nevertheless, TGF-β secreted by M2 macrophages can promote fibrosis and pathological scarring. The persistence of M2 macrophages at the injury site can result in rebound localised fibrosis and the incomplete resolution of inflammation after a tendon injury, as shown by decreased lipoxin A4 production [89]. Li et al. showed that hydroxycamptothecin (HCPT)-primed EVs or unprimed EVs reduced TGF-β1 mediated cell proliferation compared to the control group, with HCPT-EVs almost abolishing TGF-β1 mediated fibroblast proliferation [33]. This was shown histologically by reduced collagen III and α-SMA expression. Furthermore, MSC-EV modulation of macrophage polarisation can be context dependent. Systemic administration of IL-1β-primed MSCs increased macrophage M2 polarisation and increased the survival rate of murine sepsis only in the presence of miR-146a. Exosome-mediated transfer of miR-146a was shown to be necessary for the immunomodulatory response of MSCs [36]. Proteomics has also identified differences in the fibrotic potential of EVs harvested from different tissue, with tendon fibroblast-derived EVs containing a much higher amount of TGFβ1 than EVs from myoblasts and muscle fibroblasts. Nevertheless, Xu et al. reported that TGFβ is necessary for tendon-derived stem cell (TSC) exosomes to induce MSCs to secrete type I collagen [90]; this was confirmed by a rat Achilles collagenase-indued tendinopathy model, which reported a more ordered collagen fibre arrangement and increased ultimate stress and maximum loading [34].

### 4.5. Animal Models of Tendon/Ligament Injury

Animal models are crucial for modern biomedical research. They are used for diverse objectives, from testing novel pharmaceutical components to exploring new pathophysiological pathways, and are used when it is unethical or inconvenient to study on humans. However, due to the ‘unity in diversity’ concept, suggesting homology due to common evolutionary origins predicts functional similarities [91], there is a lack of guidelines and protocols for choosing the best animal model for a particular clinical context. Even humanised mice, which carry human genes or tissues, still cannot perfectly model complex human diseases like sepsis [92]. Given the enormous burden tendon and ligament injury places on society, evidence-based selection of fit-for-purpose animal models is essential.

Soslowsky et al. developed a 34-item checklist to assess the suitability of various animals for modelling human rotator cuff tendinopathy, deeming the rat the only appropriate model [93]. When running, the rat’s acromion creates an enclosed arch under which the supraspinatus tendon passes, similar to the human when lifting objects overhead [94]. However, the rat shoulder anatomy is slightly different; instead of the acromion, coracoacromial ligament and coracoid forming the arch over the supraspinatus tendon, the acromion, acromioclavicular ligament, and coracoid does so [95]. Furthermore, the use of its four limbs for locomotion makes its shoulders ‘weight bearing’, unlike the human shoulder. Nevertheless, human shoulders do bear a significant amount of stress during normal everyday activities [96]. Shoulder function in rat models can be measured by looking at ambulatory parameters. Stride length, defined as paw strike distance, is a proxy for the rat’s capacity for forward flexion and motion. In a cohort study, stride length was decreased after a multiple rotator cuff tendon detachment up to 56 days post-injury [97]. Yet, single rotator cuff tear models showed no change in stride length [98]. This suggests that the extent of rotator cuff damage correlates with the extent of functional loss, which is similar to that seen in humans, whereby those with a massive chronic rotator cuff tear pattern experience a greater loss of active shoulder range of motion [99]. Thus, stride length can be used as a valid modality to estimate the functional impact of rotator cuff pathologies.

The lack of post-operative re-tears in rat models [100] makes it appropriate to investigate biologically-based regenerative strategies for tendon/ligament repair and biomechanical strengthening of the entheses. Tendons coated with growth factors such as GDF-5 [101] and CDMP-2 [102] have shown more organized healing and superior biomechanical properties than untreated tendons, the results of which are more conspicuous in rat models due to an intact tendon–bone healing surface. Rat models have also been used for studies focusing on the biological repair of joints, rather than the anatomical repair, such as the use of reinforcement scaffolds like electrospun chitosan-coated polycaprolactone (CS-g-PCL) [103], or the reconstruction of large rotator cuff tears with acellular dermal matrix grafts [104].

Wang et al. chose to use rabbits in their rotator cuff model [35]. Although mature, the rabbits used were young, and rotator cuff tendinopathy usually occurs in the elderly. Both Wang et al. and Huang et al. created rotator cuff models via acute injury by detaching the supraspinatus tendon at the insertion on the humerus. The torn tendon was then wrapped around a silicon Penrose drain to prevent adhesion [32,35]. Furthermore, the anatomy of larger animals such as rabbits, sheep, and dogs, are not the same as humans. Sheep have no clavicle, a poorly developed acromion, and no acromioclavicular arch [105]. Nevertheless, sheep are often selected due to the similarity of their infraspinatus tendon with humans’ supraspinatus tendon, in terms of microstructure, size, and shape [106], and also because they become incapacitated if their supraspinatus is damaged [94]. The soft tissue environment also differs, with rotator cuff tendons being extra-articular [105]. This means that all repairs detach to a certain extent, with the intervening gap packed with fibrous scar tissue, which does not represent direct tendon–bone healing in humans. Also, a significant portion of the underlying joint capsule in large animals must be excised to model an intra-articular injury [107].

Large animals, with more similar tendon dimensions and biomechanics [108], are suitable for studies focusing on mechanical repair, comparing surgical repair techniques such as single-row Mason–Allen stitch and double-row medial horizontal mattress stitch at time zero [109,110], and after tendon healing [111]. The large size, the reduced ability for suture retention due to a well-aligned tendon structure, and the occurrence of re-tears in a proportion of subjects, even when post-operative activity is limited by attaching a 6-inch softball under the foot of the operated limb [112], make it more suitable for evaluating repair strategies focusing on mechanical stability and efficacy. However, like the rabbit model used by Wang et al. [35], tendons are usually acutely severed and healthy, limiting their efficacy when studying a natural degenerative process or chronically injured and diseased human tendons.

Nevertheless, large animals have also been used to model chronic rotator cuff tears. Coleman et al. reported that there is a significant increase in lipid levels in ovine rotator cuff muscles six weeks after a rotator cuff injury, leading to muscle atrophy and stiffening [113]. After tenotomy, significantly increased Myf-5 and PPARγ expression were seen [114]; these are two fat infiltration–related transcription factors found in atrophic ovine rotator cuff myocytes. This makes ovine models well suited to study the management of human rotator cuff tendinopathies with concomitant muscle pathology, since human rotator cuff pathology is also associated with fatty infiltration and progressive degenerative changes in rotator cuff muscles [115]. Coleman et al. suggested a ‘point of no return’ in rotator cuff pathologies, after which the elasticity of the muscle–tendon unit can not return to normal [113], hence frustrating any successful surgical or biological intervention. This has led some to propose that surgical management for an injured rotator cuff should be done before fatty infiltration becomes irreversible [116]. Furthermore, GFP labelling of ovine bone marrow-derived MSCs (BMSCs) confirmed the plasticity of ovine MSCs, with a transdifferentiation potential parallel to human BMSCs [117], suggesting the suitability for sheep to act as pre-clinical models for cell replacement therapy research.

In addition to rotator cuff tendons, the patella tendon is of particular interest to researchers, not only because it regularly experiences high loads, but also because it is often used in anterior cruciate ligament (ACL) grafts. Shi et al. and Yu et al. utilised a similar technique to tendon harvesting for ACL reconstruction, by creating a ‘window defect’ model whereby the central third of the patellar tendon was removed from the distal apex of the patella to the insertion of the tibial tuberosity [25,27].

Achilles tendon rupture is a frequent pathology in musculoskeletal clinics. Rats are commonly used to model Achilles tendinopathy [26,28,29,30,33,34]. Most models are created by transecting the tendon at the midpoint between the calcaneal insertion and musculotendinous junction; however, Wang et al. used a collagenase-induced Achilles tendinopathy model [34]. Recent novel approaches involve repeated Substance P injections together with Achilles tendon overuse [118], enforced downhill running in a bipedal position [119], and passive ankle exercise that simulated repetitive calf muscle contraction using an electrical stimulator [120]. The equine superficial digital flexor tendon (SDFT) is often used to mimic the human Achilles tendon, since they play similar roles during locomotion and are one of the most commonly injured tendons in both species [121]. The SDFT is a weight-bearing tendon, supports the metacarpophalangeal joint, and stores elastic energy during the stance and releases it during the swing phase, allowing efficient movement in this athletic species, much like the Achilles tendon [122]. Moreover, with its relatively long life-span, equine ageing best mirrors that of humans, and is good for studying ageing-induced tendon degeneration. The frequent incidence of equine SDFT injury, with a 23% prevalence [123], contributed to a ‘One Health One Medicine’ notion, suggesting that diseases in humans and animals (especially mammals) are pathophysiologically alike and require similar management plans [124]. Hurtig et al. suggested that in order to achieve a timely transition from lab experiments to clinical use, it is best to initially utilise small lab animals, followed by pilot studies on large animals, given that they have closer immunophysiological properties to humans and are large enough for gait analysis and arthroscopic interventions [125].

It is accepted that no one animal model of tendon or ligament injury can perfectly replicate human pathology. Oreff et al. calculated the Mahalanobis distances, a non-dimensional measure of dissimilarity, of four model species to humans: mouse, rat, horse, and sheep, and found that the species that best matched humans depended on which functional group of genes were analysed [126]. Horses had the shortest Mahalanobis distance to humans in terms of matrix remodelling proteinases, suggesting that horses are most suitable for assessing ECM production and tendon healing. However, rat tenocytes best resembled humans in terms of inflammatory mediators; furthermore, under healthy conditions, rat tenocytes had the shortest Mahalanobis distance with regards to overall gene expression [126], bringing into question Hurtig et al.’s proposition that large animals have more similar properties to humans.

### 4.6. In Vivo Findings

No standardised histological or immunohistochemical tests were used across the studies. Gissi et al. utilised a semi-quantitative histomorphometric scoring system [29] that was modified from that proposed by Soslowsky et al., Svensson et al., and Cook et al. [93,127,128]. This considered four parameters, namely cartilage formation, vascularity, cellularity, and fibre structure. Yu et al. defined their own unique histological scoring system with six parameters [27]. Both Yao et al. and Li et al. utilised the same histological adhesion scoring system and histological healing scoring system; the former took into account the percentage of adhesion area on the tendon surface, and the latter was based on whether or not the collagen fibres looked smooth and regular [30,33]. Although many histological scoring systems overlap in the parameters they look for, they nevertheless present difficulty in pooling data, precluding any meta-analysis.

The organization of fibrous connective tissue within the defect site was evaluated using a parallel fibre alignment scoring method [129]. While the histological grading methods have been proven in vitro to be hallmarks of better healing, there are no studies directly linking histological evidence of healing to mechanical strength. Yao et al. showed that the exosome treated group had significantly decreased COL III, α-SMA, p-p65, and COX2 expression; however, these favourable immunohistochemical tests did not translate into improved maximal tensile strength [30]. Similarly, Chamberlain et al. showed that exosome treatment increased type I and type III collagen production within the granulation tissue; however, it did not significantly improve mechanical function [31]. Li et al. demonstrated that both unprimed EVs treatment and HCPT-EVs treatment dramatically lowered the adhesion grade of the tendon; however, this did not increase the maximal tensile strength of the regenerated tendon [33].

Biomechanical properties are the ultimate index for evaluating tendon–bone healing. Although the animal models varied by species, joint and time post-EV delivery, when the tendons were harvested for biomechanical testing, the methods used were very similar across all studies (Table 4). All loaded tendons into universal testing machines and stretched them to failure at a constant speed. Wang et al. [35] removed sutures prior to biomechanical testing, allowing for the accurate testing of the repaired tendon segment. Challenges exist with biomechanical testing of tendons. Many lab animals are quadrupeds and subject their tendons to different magnitudes of load than their human counterparts, making it difficult to replicate the pathology seen clinically. For all included studies, mechanical properties were assessed in vitro after tendon dissection and removal of surrounding tissue. Ex vivo testing of viable tendon samples provides information concerning the initial cellular and matrix response to loading but still cannot take into account in vivo healing. The in vitro testing methods discussed thus far can provide very controlled loading conditions, but cannot mirror the complexity of the native tissue environment. In vivo, so-called animal overuse models, overcome this by enabling the consideration of cellular responses within the native tissue environment. However, the degree of reproducibility of loading can be harder to control [130].

In vitro limitations include considerable issues associated with the gripping of the two tendon ends, which precludes the testing of the tendon-to-bone attachment. Testing the strength of the tendon-to-bone attachment is of considerable clinical value as rotator cuff re-tears usually develop at the junction between the tendon and the bone [131,132]. Although there are limitations with the biomechanical models used, the data reviewed is useful and all included studies faced the same limitations, making results comparable, with a trend towards improved maximum force, elastic modulus, and strength in EV treated groups.

Recommendations for future work would be the ex vivo testing of biomechanics using novel 3D-printed fixtures that exactly match the anatomies of the humerus and calcaneus to mechanically test supraspinatus tendon and Achilles tendon, respectively. Kurtaliaj et al.’s new approach eliminated artifactual gripping failures (e.g., growth plate failure rather than in the tendon), decreased overall testing time, and increased reproducibility [132]. Furthermore, it is challenging to generate models which mimic the cumulative damage seen in age or overuse related tendinopathy. The mechanically and chemically induced models used in these studies better model acute injuries. A representative pathophysiological tendon model can be established by combining mild overstimulation for a longer period of time (e.g., three weeks), mimicking the chronic situation, and acute extreme overloading and/or scratch, representing the acute injury [133].

Three studies reported no significant difference in biomechanical properties between EV-treated and control groups [30,31,33]. Yao et al. did not find a difference in terms of biomechanical strength in the exosome-treated group [30]. The maximum tensile strength was evaluated at three weeks following exosome treatment and primary repair with a 6-0 polypropylene suture. Li et al. also repaired the Achilles tendon using the 6-0 polypropylene suture and harvested it after three weeks [33]. Chamberlain et al. utilised a rat medial collateral ligament (MCL) injury model, and performed biomechanical testing 14 days post-injury [31]. The MCL was not subject to primary repair in this model. Based on these findings, we postulate that at three weeks post-injury, the tensile strength of the tendon repair was predominantly due to the tensile strength of the 6-0 polypropylene suture repair. Wang et al. found that ultimate stress and maximum loading were significantly increased in the exosome treated group compared with injury; however, they did not compare TSCs to TSC-derived EVs regarding biomechanics [34]. Only TSC versus phosphate-buffered saline (PBS) and EVs versus PBS were compared, raising the possibility of reporting bias. No included studies reported stem cells versus EVs. For those studies which did report EVs having a positive impact on the biomechanics of ligament/tendon, the shortest follow-up time post-injury was four weeks. This might suggest that the effect of EVs on healing is mostly realised beyond the four-week point. Studies with a larger sample size and animals sacrificed at multiple time points to allow for biochemical and biomechanical studies need to explore this hypothesis and plot a timeline for histological and biomechanical improvement.

### 4.7. Limitations

One major limitation of this systematic review was the heterogeneity between studies regarding outcome measures reported. The qualitative and quantitative evaluation of tendon and ligament repair included macroscopic, histological, biochemical, and biomechanical analysis which differed widely between studies. Only six studies presented macroscopic analysis; all apart from Yao et al. [30] were qualitative in nature and therefore subject to observer bias, making fair comparison between studies difficult. Similarly, although many histological scoring systems were used, and while there was overlap in the parameters they look for, this heterogeneity precluded any meta-analyses.

The majority of the analysis time points were done prior to four weeks after MSC-EV application. This does not allow for long–term evaluations, which is especially important for assessing animal mobility, whose evaluation can only be done after tissue integration over time has taken place. Only Wang et al. performed longer-term analysis, at 18 weeks after exosome injection [35].

The majority of the studies in this review were low risk, as determined by the SYRCLE RoB assessment tool. Ten studies in this review had low bias; only Huang et al. had some concern towards the risk of bias [32], with the main contributors being blinding and detection bias. Nevertheless, only seven studies examined the specific cell surface antigens of MSCs via flow cytometry, with four of them further performing a trilineage-induced differentiation assay to identify the differentiation potential of MSCs. All studies quantified EV dimensions and identified EV surface biomarkers, as stated in the Minimal Information for Studies of Extracellular Vesicles (MISEV) criteria [23]. We suggest that all future studies define their MSC-EV populations physically, biochemically, and functionally by quantifiable features, as recommended by the International Society for Cellular Therapy (ISCT) [22], utilise objective quantifiable methods for macroscopic and histological analysis, and standardise MSC-EV delivery methods, based on the animal model used, and the ideal volume and/or cell number for a given defect size.

## 5. Conclusions

Tendinopathy is a common disorder that results in a significant disease burden. Regenerative approaches via tissue engineering are a promising option, especially novel cell-free therapies utilising MSC-EVs, which have been shown to be effective in in vitro studies. Randomised studies in suitable animal models that mimic human disease are necessary before progression to human trials. In this review, all included in vivo studies reported better tendon/ligament repair following MSC-EV treatment, but not all found improvements in every parameter measured. Although biomechanical properties are very relevant for assessing tendon and ligament healing, this was not consistently assessed. Even if it was assessed, evidence linking biomechanical alterations to functional improvement was weak; studies are needed that rigorously examine the underlying mechanisms for the enhancement of biomechanical properties after MSC-EV treatment. The progression of promising preclinical data to achieve successful clinical market authorisation remains a bottleneck. One hurdle for progress to the clinic is the transition from small animal research to advanced preclinical studies in large animals to test for the safety and efficacy of products. However, it is likely that there will be translational questions not completely answered by animal models as co-morbidities (e.g., obesity, smoking) will be challenging to model. Nevertheless, the studies in this review have showcased the safety and efficacy of MSC-EV therapy for tendon/ligament healing, by attenuating the initial inflammatory response and accelerating tendon matrix regeneration, providing a basis for potential clinical use in tendon/ligament repair.

We have given a rationale for the further development of a ready-to-use, cell-free, MSC-based approach that is highly effective for tendon and ligament repair. Future studies should focus on forming a link between histological, biochemical, and functional outcomes, quantify macroscopic assessment of tendon repair, and standardise histological scoring systems, whilst adhering to ISCT and MISEV guidelines for MSC-EV research.

## Figures and Tables

**Table 1:** Characterisation of MSCs.

Article	Source	Cell Origin	Cell Treatment	MSC Verification
Shi et al. [25]	Sprague–Dawley rats	Bone marrow cells from the femur and tibia	Cultured in α-MEM containing 10% FBS until third to fifth passage. MSCs were then cultured in Mesen Gro MSC medium.	Flow cytometry: CD44, CD90 +ve; CD11b, CD34 −ve
Shen et al. [26]	NGL transgenic reporter mice and Scleraxis–GFP tendon reporter mice	Adipose tissue (subcutaneous fat)	Cultured in 10% FBS, 100 unit/mL penicillin, and 100 μg/mL streptomycin in α-MEM	Flow cytometry: CD29, CD44, CD90 +ve
Yu et al. [27]	Sprague–Dawley rats	Bone marrow cells from the femur and tibia	Cultured in α-MEM containing 10% FBS, 100 U/mL penicillin and 100 mg/mL streptomycin until second passage. Cells were then cultured in an exosome-depleted medium.	Trilineage differentiation (adipocytes, osteoblasts, and chondrocytes); Flow cytometry: CD44, CD90 +ve
Shi et al. [28]	C57BL/6 male mice	Bone marrow cells from the femur	Cultured in α-MEM supplemented with 20% FBS, 1% penicillin, and streptomycin until third to fifth passage.	Flow cytometry: CD44, CD90, Sca-1 +ve; CD34, CD45 −ve
Gissi et al. [29]	Lewis rats	Bone marrow cells from the femur and tibia	Cultured in MesenCult Basal Medium, supplemented with penicillin/streptomycin (100 U/mL–100 μg/mL) and 10% FBS until second passage.	Trilineage differentiation (adipocytes, osteoblasts, and chondrocytes); Flow cytometry: CD29, CD44, CD90 +ve; CD45, CD34 −ve
Yao et al. [30]	Human	Umbilical cord	Cultured in α-MEM mixed with 10% FBS until 3rd to 5th passage.	Not done.
Chamberlain et al. [31]	Human	Bone marrow cells	Cultured in α-MEM mixed with 10% FBS, 1× nonessential amino acids, and 4 mM l-glutamine until 4th to 6th passage.	Not done.
Huang et al. [32]	Sprague–Dawley rats	Bone marrow cells from the femur and tibia	Cultured in standard media comprising DMEM supplemented with 10% FBS and 1% double antibiotics (streptomycin + penicillin).	Trilineage differentiation (adipocytes, osteoblasts, and chondrocytes); Flow cytometry: CD44, CD73, CD90, CD105 +ve; CD34 −ve
Li et al. [33]	Human	Umbilical cord	Cultured α-MEM supplemented with 10% FBS and 1% penicillin/streptomycin	Not done.
Wang et al. [34]	Sprague–Dawley rats	Tendon stem cells from the Achilles tendon	Cultured in DMEM containing 10% FBS, 100 U/mL penicillin and 100 mg/mL streptomycin until day 7. Cells were then trypsinised with EDTA.	Trilineage differentiation (adipocytes, osteoblasts, and chondrocytes). Immunostaining; CD44, CD90 +ve; CD3, CD34 −ve, staining by Sirius red, Oil Red O and Alizarin red respectively.
Wang et al. [35]	Human	Adipose tissue (subcutaneous fat)	Cultured in serum-free medium (OriCell). The second and third passages were used for ASCs-Exos isolation.	Not done.

α-MEM = alpha-modified minimum essential medium; FBS = foetal bovine serum; DMEM = Dulbecco’s Modified Eagle Medium; NGL = NF-κB-GFP-luciferase; MSCs = mesenchymal stem cells; ASCs = adipose-derived mesenchymal stem cells.

**Table 2:** Characterisation of EVs.

Article	EV Purification	EV Dimensions	EV Biomarkers	**Imaging**	**Active Component**
Shi et al. [25]	Conditioned media concentrated by several centrifugation and ultracentrifugation, then passed through a 0.22 μm filter.	qNano Gold: 70–600 nm in diameter.	CD9, CD63, HSP70	TEM	Not assessed
Shen et al. [26]	“Conditioned media concentrated by several centrifugation and ultracentrifugation, then passed through a 0.22 μm filter.	qNano Gold: Mode diameter of iEVs and EVs were 108 ± 2 nm and 113 ± 3 nm, respectively.	CD9, CD63	TEM	Not assessed
Yu et al. [27]	Conditioned media concentrated by several centrifugation and ultracentrifugation.	NTA with ZetaView: 101.1 ± 50.6 nm in diameter	CD9, ALIX, TSG101	TEM	Not assessed
Shi et al. [28]	Conditioned media concentrated by several ultracentrifugation steps.	NTA with ZetaView: average diameter 120.3 nm; peak of size distribution 127.1 nm	CD81, TSG101, CD9	TEM	Not assessed
Gissi et al. [29]	Conditioned media concentrated by several centrifugation and ultracentrifugation.	AFM: 40–280 mm in diameter, with a peak at 80 mm.	Annexin XI, Annexin V and TSG-101 +ve; GM-130 −ve	AFM	Pro-collagen1A2 and MMP14
Yao et al. [30]	Conditioned media concentrated by several ultracentrifugation steps.	NTA with ZetaView: 30–200 nm, average of 131 nm.	CD9, CD63, ALIX, TSG101	80 kV electron microscope	MicroRNA-21-3p
Chamberlain et al. [31]	Conditioned media concentrated by differential centrifugation and ultracentrifugation steps.	qNano Gold: 61–121 nm	CD146, CD29, CD44, CD63, CD81, and CD105.	TEM	Not assessed
Huang et al. [32]	Conditioned media concentrated by differential centrifugation and ultracentrifugation steps.	TEM: 30–150 nm	CD9, CD63, and CD81	TEM	Not assessed
Li et al. [33]	Conditioned media concentrated by differential centrifugation and ultracentrifugation steps.	TEM: 30–150 nm	CD9, CD63, ALIX, TSG101	TEM	Not assessed
Wang et al. [34]	Conditioned media concentrated by differential centrifugation and ultracentrifugation steps.	TEM: 40–200 nm	CD63 and CD81	TEM	Not assessed
Wang et al. [35]	Conditioned media concentrated by several centrifugation and ultracentrifugation.	qNano Gold: 50–150 nm	CD9, CD63, TSG-101 +ve, GM130 −ve	TEM	Not assessed

EV = extracellular vesicle; NTA = Nanoparticle tracking analysis; TEM = Transmission electron microscopy; ATM = Atomic force microscopy.

**Table 3:** Characteristics of animal models.

Article	Method of Delivery	Animal Model	Number Used	Animal Age	Animal Weight	Animal Gender	Follow-up	Number per Experimental Group
Shi et al. [25]	10 µL of fibrin containing 25 µg BMSC-EVs was applied around the injury site.	Sprague–Dawley rats Central 1/3 of patellar tendon was removed from the distal apex of the patella to the insertion of the tibial tuberosity.	48	N/A	N/A	Male	2 weeks (immunohistochemistry analysis) 4 weeks (histological analysis)	(1) BMSC-EVs group (n = 16) (2) Fibrin group (n = 16) (3) Control group, left untreated (n = 16)
Shen et al. [26]	ASC-EVs were loaded to the surface of a collagen sheet, that was cut into strips, each containing 5–6 × 10^9^ EVs. Applied around the defect site.	NGL transgenic reporter mice. Right Achilles tendon 2/3 transection at midpoint level between calcaneal insertion and musculotendinous junction.	32	3–4 months	27 ± 5 g	Male and female	7 days	(1) Collagen sheet loaded with EVs from naïve ASCs (n = 11) (2) Collagen sheet loaded with EVs from IFNγ-primed ASCs (n = 10) (3) Collagen sheet only (n = 11)
Yu et al. [27]	5 µL of BMSCs-exos (4 µg/µL) was mixed with 1 µL thrombin (500 IU/mL) and 4 µL fibrinogen (50 mg/mL), injected into the defect site.	Sprague–Dawley rats Central 1/3 of the patellar tendon (0.8 mm in width) was removed from the distal apex of the patella to the insertion of the tibial tuberosity.	52	Adult	200 g	Male	1 week, 2 weeks, and 4 weeks (macroscopic and histological examination) 4 weeks (mechanical test)	(1) Fibrin-exos (fibrinogen, thrombin, and exosomes injected) (n = 26) (2) Fibrin-vehicle (fibrinogen, thrombin, and PBS injected) (n = 26)
Shi et al. [28]	Exosomes were mixed with hydrogel before implantation into the cut Achilles tendon.	C57BL/6 mice. The Achilles tendon was cut off above the calcaneus.	90	8 weeks	20–25 g	Male	7 days	(1) Control group (n = 30) (2) Hydrogel group (n = 30) (3) Hydrogel + exosome group (n = 30)
Gissi et al. [29]	50 μL of PBS was injected locally with either EV_L_ (2.8 × 10^12^) or EV_H_ (8.4 × 10^12^).	Lewis mice. Bilateral Achilles tendon defect 2 mm in diameter was made in each animal.	16	Adult	180–200 g	Male	30 days	(1) PBS alone (control group) (n = 4) (2) rBMSCgroup: 4 × 10^6^ cells (n = 4) (3) EV_L_ group: 2.8 × 10^12^ EVs (n = 4) (4) EV_H_ group: 8.4 × 10^12^ EVs. (n = 4)
Yao et al. [30]	Injected subcutaneously around injury site with HUMSC-Exos (200 μg) dissolved in PBS and an equal volume of PBS (50 μL).	Sprague-Dawley rats. The Achilles tendon was cut in the middle.	60	Adult	200–250 g	Male	3 weeks	(1) Sham group (n = 20) (2) HUMSC-Exos group (n = 20) (3) PBS group (n = 20)
Chamberlain et al. [31]	Injected 1 × 10^9^ exosomes to MCL transection site.	Wistar rats Bilateral MCL transection at its midpoint.	10	Adult	300–350 g	Male	14 days	(1) Exosomes (n = 5) (2) PBS (control) (n = 5)
Huang et al. [32]	200 μg of BMSC-Exos precipitated in 200 μL of PBS was injected into the tail vein.	Sprague-Dawley rats. 2 mm of the distal tendon of the supraspinatus was cut off.	54	4 weeks	70–100 g	Male	4 weeks	(1) BMSC-Exos group (n = 27) (2) PBS (control) (n = 27)
Li et al. [33]	HCPT-EVs were both subcutaneously injected at the injury site at a dose of 200 μg.	33 Sprague-Dawley rats The Achilles tendon was transected in the middle and repaired using the 6-0 polypropylene suture.	33	Adult	250–300 g	Male	3 weeks	(1) PBS (n = 11) (2) Unprimed EV injection (n = 11) (3) HCPT-EV injection (n = 11)
Wang et al. [34]	20 μL of exosomes (486.3 μg/mL) was injected into the Achilles tendon injury site twice a week.	18 male Sprague-Dawley rats Rats were injected with 30 µL type I collagenase solution (10 mg/mL) into both Achilles tendons.	18	8 weeks	200–250 g	Male	4 weeks	(1) PBS (control) (n = 6) (2) Injury group with TSCs (n = 6) (3) Injury group with exosomes (n = 6)
Wang et al. [35]	10^11^ ASC-Exos suspended in 20 µL of saline were injected at the injury site of the supraspinatus muscle.	Rabbits: Bilateral rotator cuff tear model. The supraspinatus tendon was detached at the insertion on the humerus. The torn tendon was wrapped with a silicon Penrose drain to prevent adhesion.	35	4 months	3.3 ± 0.3 kg	Male	18 weeks	(1) Repair + saline (n = 7) (2) Repair + ASC-Exos (n = 7) (3) Sham surgery (n = 14) (4) Fatty infiltration assay (n = 7)

EVs = extracellular vesicles; MSC = mesenchymal stem cells; BMSC = bone marrow-derived multipotent mesenchymal stromal cells; rBMSCs = rat bone marrow-derived MSCs; ASCs = adipose-derived mesenchymal stem cells; PBS = phosphate-buffered saline; HUMSC = human umbilical mesenchymal stem cells; NGL = NF-κB-GFP-luciferase; HCPT = hydroxycamptothecin.

**Table 4:** In vivo Findings.

Article	Macroscopic Appearance	Imaging and Histology	Biochemical Analysis	Biomechanical Analysis
Shi et al. [25]	Not undertaken.	Regularly aligned and compact collagen fibres. Fibre alignment score of 2 (50% to 75% parallel fibre alignment) Increased tendon cell proliferation, especially after treatment with BMSC-EVs at 20 μg/mL.	Elevated number of cells expressing CD163, IL-4, and IL-10 in the BMSC-EVs group. Reduced number of cells expressing IFNγ, IL-1B, IL-6, and CCR7 in the BMSC-EVs group. Increased expression of SCX, TNMD, COL1a1, and COL3a1 in the BMSC-EVs group. Increased gene expression of collagen type I in the BMSC-EVs group. Reduced cleaved caspase 3 signals in the BMSC-EVs group.	Not done
Shen et al. [26]	Not undertaken.	Significant reductions of NF-κB activity in iEV-treated tendons compared to untreated tendons, but little reduction after EV treatment. Lower gap-rupture rate. iEV-treated tendons exhibited more collagen staining at the site of tendon injury than did untreated and EV-treated tendons.	Expression levels of inflammatory genes Ifng, Nos2, Tnf, Il6, Mmp1, Col1a1 and Col3a1 increased after injury. Treatment with iEV but not EVs significantly reduced Il1b and Ifng expression. Treatment with both iEVs and EVs significantly attenuated the Mmp1 expression, increased Col2a1 and Sox9 expression. iEV but not EV treatment further increased both Col1a1 and Col3a1 expression.	Not done
Yu et al. [27]	The exosome-treated group showed improved integration of the healing tissue with the host tendon at week 2, and showed a more approximate appearance (colour and transparency) to the native tendon at week 4.	More deposition of extracellular matrix type I collagen at week 2. At week 4, cell density and alignment in the defect region of the exosome-treated group were much closer to the native tendon. Lower histological score in the exosome-treated group at week 4 (suggesting better tendon regeneration).	The exosome-treated group showed much higher expression of Col I and Tnmd. The ratio of proliferating CD146^+^ TSPC to total CD146^+^ cells was 1.73–fold higher in the exosome-treated group 1–3 days post-injury, but not afterwards.	**Method**: The tendon tissue was put on a universal tensile testing machine (AGS-X, SHIMADZU), cyclically elongated for 20 cycles, and stress at failure was calculated as ultimate load divided by cross sectional area. **Results**: The stress at failure of the healing tendons and modulus were 1.84–fold and 1.86–fold higher in the exosome-treated group compared to the control.
Shi et al. [28]	Initially, there was less scar hyperplasia in the hydrogel + exosome group than in the control and hydrogel groups.	In the hydrogel+exosome group, a transition structure similar to tendon–bone interface was seen, chondrocyte numbers increased and were tightly arranged, collagen tissues were arranged orderly.	M2 macrophages (Arg1+) increased and M1 macrophages (iNOS+) decreased in the hydrogel+exosome group. The hydrogel+exosome group showed decreased IL-1β and IL-6 and increased IL-10 and TGF-β1. Reduced TUNEL-positive apoptotic cells, increased CD146-positive stem cells in the hydrogel+exosome group. Increased gene expression level of collagen II in hydrogel+exosome group.	**Method**: The tissue was loaded into a universal testing machine, preloaded with small tension, then stretched to failure at a constant speed. **Results**: Maximum force, elastic modulus, and strength in the hydrogel+exosome group were higher than hydrogel and control groups, but no significant difference with the normal group. No significant difference in stiffness between groups.
Gissi et al. [29]	Not undertaken.	Lower overall histomorphometric score in EV_H_ group than rBMSC and EV_L_ groups. Only the cellularity sub-score in the EV_H_ group was higher than the control group. Overall suggests a better restoration of tendon architecture, optimal alignment of tendon fibres and blood vessels in the EV_H_ group.	The EV_H_ group had a more favourable collagen ratio: higher collagen type I and lower collagen type III than rBMSC, EV_L_ and control groups.	Not done.
Yao et al. [30]	The degree of adhesion of tendon tissue with HUMSC-Exos application was lower than in the PBS and sham groups. Lower adhesion grade score in the HUMSC-Exos group.	Hyperproliferative adhesion tissue, and degree of inflammatory infiltration were lower in the HUMSC-Exos group compared to the PBS and sham groups. The HUMSC-Exos group had the lowest histological adhesion score. The histological healing score was not statistically different among the three groups. The HUMSC-Exos group had the least collagen deposition.	HUMSC-Exos significantly decreased COL III, α-SMA, p-p65, and COX2 expression.	**Method**: Tendon tissue fixed to a biomechanical analyser (Instron 8841 DynaMight axial servo hydraulic test system), stretched at constant speed until the tendon broke. **Results**: No significant difference in maximum tensile strength between the three groups.
Chamberlain et al. [31]	Treatment with exosomes significantly reduced scar formation 14 days post-injury compared to the control.	Exosome treatment increased type I and type III collagen production within the granulation tissue. Exosome treatment improved collagen organisation.	IHC analysis CD68, CD163, CD31, and α-smooth muscle actin levels, to identify M1 and M2 macrophages; no changes elicited in M1 and M2 macrophages.	**Method**: Mounted in the mechanical testing machine, preconditioned with cyclic preloading, then pulled to failure at constant strain rate, with parameters recorded. **Results**: Treatment with exosomes did not significantly improve mechanical function.
Huang et al. [32]	Not done.	Angiography showed that BMSC-Exos promoted angiogenesis around the rotator cuff endpoint.	BMSC-Exos promoted the expression of CD31 and endomucin. BMSC-Exos significantly reduced the serum levels of TNF-α, IL-1β, IL-6, and IL-8. BMSC-Exos promoted Col I and Col II expression, expression of Sharpey’s fibres and proteoglycan at the tendon-bone interface.	**Method**: Freshly excised tissue was immediately placed in paraformaldehyde solution, then loaded onto biomechanical tester, with a constant displacement distance being applied until failure. **Results**: BMSC-Exos increased the maximum breaking load and stiffness.
Li et al. [33]	Macroscopic observation showed that both HCPT-EVs and unprimed EVs effectively attenuated tendon adhesion to peri-tendinous tissues.	Histological adhesion scores based on histological findings. The results showed that both unprimed EVs treatment and HCPT-EVs treatment dramatically lowered the adhesion grade of the tendon. Comparing the scores achieved by HCPT-EVs with unprimed EVs showed a tendency toward decreasing, although it was not significant. The histological healing score was significantly lower in the group treated with HCPT-EVs than with unprimed EVs.	HCPT-EVs more effectively decreased myofibroblast activation induced by TGFβ after tendon injury, as demonstrated by weaker WB staining of both COL III and α-SMA in unprimed EVs or HCPT-EVs. qRT-PCR analysis suggested that unprimed EVs and HCPT-EVs suppressed COL III and α-SMA at the transcription level and displayed a larger decreasing trend after HCPT-EVs administration than with unprimed EVs.	**Method**: Both ends of the tissue are clamped in a tensile testing system, and stretched at a constant speed of 10mm/minute until rupture. The maximum tensile force was recorded. Five samples from each group were used for analysis. **Results**: The maximal tensile strength of the regenerated tendon remained the same among the three groups.
Wang et al. [34]	Not done.	The arrangement of collagens in the exosomes group was more uniform than that of the injury group.	TSCs injection and exosomes injection significantly decreased matrix metalloproteinases (MMP)-3 expression, increased expression of tissue inhibitor of metalloproteinase-3 (TIMP-3) and Col-1a1	**Method**: The two bony ends of the tendon were fixed on a custom-made testing jig with two clamps. No further details are available. **Results**: Ultimate stress and maximum loading were significantly increased in the exosome treated group compared with injury. No TSC vs exosome comparison was made.
Wang et al. [35]	Fatty infiltration was significantly higher in rabbits with rotator cuff tear than those receiving sham surgery (confirming the establishment of a rotator cuff tear model).	Few inflammatory cells were present in the ASC-Exos group than in the saline group Cellularity and vessel numbers at tendon-bone interface of the ASC-Exos group were significantly lower than those in the saline group. The fibrocartilage area in the ASC-Exos group was significantly greater than in the saline group. More abundant collagen II and tenascin-C appeared in the ASC-Exos group than in the saline group.	Lower expression of CD31 in the ASC-Exos group than the saline group, attributed to the maturation of tiny capillaries into proper blood vessels.	**Method**: Tendons harvested and loaded into clamping device. Specimens were preloaded to 5 N for 5 min, with 10 cycles of preconditioning (5 N to 30 N at a rate of 15 N/s). Then, each specimen was loaded to failure using a 0.5-mm/min uniaxial tension. Ultimate load to failure, stiffness, and stress were calculated according to the load-elongation curve. **Results**: Mean ultimate load to failure of the ASC-Exos group (132.7 ± 10.3 N) significantly greater than that in the saline group (96.0 ± 9.8 N) though lower than in the sham surgery group (162.2 ± 12.1 N).

EVs = extracellular vesicles; iEV = IFNγ primed EVs; MSCs = mesenchymal stem cells; ASCs = adipose-derived mesenchymal stem cells; TSCs = tendon stem cells; TSPCs = tendon stem/progenitor cells; BMSC = bone marrow-derived multipotent mesenchymal stromal cells; rBMSCs = rat bone marrow-derived MSCs; HUMSC = human umbilical mesenchymal stem cells; IHC = immunohistochemistry.

## Data Availability

All data generated or analysed during this study are included in this published article (and its supplementary information files).

## Supplementary Materials

The following are available online at https://www.mdpi.com/article/10.3390/cells10102553/s1, Table S1: Search Strategy, Table S2: Inclusion and Exclusion Criteria.
