# Supplementary material for: Mesenchymal Stem Cell-Derived Extracellular Vesicles in Tendon and Ligament Repair—A Systematic Review of In Vivo Studies
Source: Cells. 2021 Sep 27;10(10):2553. doi: 10.3390/cells10102553 (PMC8533909; doi:10.3390/cells10102553)
Supplement: Supplementary file 1 [file cells-10-02553-s001.zip › cells-1340860-supplementary.pdf]

Supplementary Table S1: Search Strategy

**1**	mesenchymal stem cell*.mp
**2**	multipotent stromal cell*.mp
**3**	multipotent stem cell*.mp
**4**	mesenchymal stromal cell*.mp
**5**	multipotent mesenchymal stromal cell*.mp
**6**	extra-cellular vesicle*.mp
**7**	extracellular vesicle*.mp
**8**	exosom*.mp
**9**	microvesicle*.mp
**10**	ectosom*.mp
**11**	microparticle*.mp
**12**	tendon*.mp
**13**	ligament*.mp
**14**	articular ligament*.mp
**15**	connective tissue*.mp
**16**	elastic fibre*.mp
**17**	collagen fibre*.mp
**18**	strain*.mp
**19**	tear*.mp
**20**	1 OR 2 OR 3 OR 4 OR 5
**21**	6 OR 7 OR 8 OR 9 OR 10 OR 11
**22**	12 OR 13 OR 14 OR 15 OR 16 OR 17 OR 18 OR 19
**23**	20 AND 21 AND 22

Supplementary Table S2: Inclusion and Exclusion Criteria

	Inclusion Criteria	Exclusion Criteria
**Population**	- In vivo experiments on animal models of tendon and ligament injury.<br>- Animals of all ages, sexes, and species.	- Animals with co-morbidities<br>- Animal models of periodontal ligament injury
**Intervention**	- Treatment with extracellular vesicles derived from human or animal derived mesenchymal stem cells.<br>- Any dosage, timing, frequency, location of extracellular vesicle application will be included.<br>- Tendon and ligament injury induced by surgical or non-surgical means	None
**Control**	- Studies with at least one control arm, whereby control medium or saline was injected into animal models.	- Studies that alter the biological properties of the animal model by injecting other chemicals.
**Outcomes**	- Source and cell origin of mesenchymal stem cells (MSCs).<br>- How the cell was treated/cultured to obtain MSCs.<br>- MSC characterisation using flow cytometry/western blotting.<br>- How the MSCs were treated to obtain extracellular vesicles (EVs).<br>- Dimensions of EVs obtained, EV surface biomarkers.<br>- How ligament and tendon damage was induced in the animal model.<br>- Method and quantity of EV delivery into the animal model.<br>- Age, weight, gender of animal model, and total number used.<br>- Follow-up time.<br>- Experimental groups in each study.<br>- Macroscopic appearance of animal model after EV delivery.<br>- Imaging and histological results after EV delivery.<br>- Biochemical analysis.<br>- Biomechanical analysis.	- Exclude studies with no description of MSCs that were harvested, or no description of EVs, or no description of in vivo findings in the animal model after EV delivery.
**Study Design**	- Randomised control trials, case-control studies published in any year.<br>- English language studies.<br>- Studies with any length of follow-up.	- Exclude studies not written in or translated to English.<br>- Exclude conference abstracts, dissertations, letter to the editor, review articles.<br>- In vitro, ex vivo, in silico experiments.<br>- In vivo studies in humans.
